# Biomolecular functionalization for enhanced cell–material interactions of poly(methyl methacrylate) surfaces

**DOI:** 10.1093/rb/rbv014

**Published:** 2015-08-07

**Authors:** Xavier Punet, Rodolphe Mauchauffé, José C. Rodríguez-Cabello, Matilde Alonso, Elisabeth Engel, Miguel A. Mateos-Timoneda

**Affiliations:** ^1^Biomaterials for Regenerative Therapies Group, Institute for Bioengineering of Catalonia (IBEC), Barcelona 08028, Spain,; ^2^CIBER en Bioingenería, Biomateriales y Nanomedicina (CIBER-BBN), Spain,; ^3^G.I.R. Bioforge, Universidad Valladolid (UVA), Valladolid 47011, Spain and; ^4^Department of Material Science and Metallurgical Engineering, Technical University of Catalonia (UPC), Barcelona 08028, Spain

**Keywords:** cell adhesion, elastin-like recombinamer, poly(methyl methacrylate), PMMA, RGD peptide, surface modification

## Abstract

The integration of implants or medical devices into the body tissues requires of good cell–material interactions. However, most polymeric materials used for these applications lack on biological cues, which enhanced mid- and long-term implant failure due to weak integration with the surrounding tissue. Commonly used strategies for tissue–material integration focus on functionalization of the material surface by means of natural proteins or short peptides. However, the use of these biomolecules involves major drawbacks such as immunogenic problems and oversimplification of the constructs. Here, designed elastin-like recombinamers (ELRs) are used to enhance poly(methyl methacrylate) surface properties and compared against the use of short peptides. In this study, cell response has been analysed for different functionalization conditions in the presence and absence of a competing protein, which interferes on surface–cell interaction by unspecific adsorption on the interface. The study has shown that ELRs can induce higher rates of cell attachment and stronger cell anchorages than short peptides, being a better choice for surface functionalization.

5th China-Europe Symposium on Biomaterials in Regenerative Medicine (CESB 2015) Hangzhou, China April 7–10, 2015

## Introduction

Poly(methyl methacrylate), PMMA, was first used into clinics, as dental device, for the fabrication of complete denture bases [1]. Due to its biocompatible characteristics, its reliability, ease manipulation and low toxicity, the usage of PMMA was extended into other medical applications such as bone cements [2], contact and intraocular lenses [3], screw fixation in bone [4], skull defects reparation [5], stabilization of vertebrae in osteoporotic patients [6], intradermal fillers [7] and mandibular reconstruction [8]. Although numerous new biomaterials have appeared on the market, the versatility and reliability of PMMA cause it to remain a popular and frequently used material [9].

For many of these applications, such as mandibular reconstruction or intraocular lenses, a good cell–biomaterial interaction is paramount for the integration of the implant, and the stability of the fixation on the interface or the prevention of cataract, respectively. However, PMMA shows weak or null interactivity against tissue cells, which promotes the formation of fibrotic tissue [10, 11]. The formation of fibrotic tissue around the implant is known to be one of the most common causes of implant integration failure, which compromises its long-term viability [11].

To enhance tissue–biomaterial interaction, and therefore implant integration, many strategies have been used, such as modification of surface properties. A common way to improve surface properties is done by chemical surface modification, which can be obtained through the addition of functional chemical groups or bioactive molecules [12, 13]. The latter option, the addition of bioactive molecules, has proved to present stronger and more specific interaction towards cells, and has been widely exploited to enhance cell–biomaterial interactions [13]. Especially noteworthy is the use of RGD (arginine–glycine–aspartic acid) amino acidic sequence, which is a peptide sequence that has been deeply studied because of its cell adhesion properties [14].

The use of amino acidic sequences that interact with integrins (adhesive cell membrane proteins) has clearly been proved to be useful on bioengineering [14]. However, there is great discussion about which is the best way to add these biological cues onto material surfaces, through the grafting of natural proteins or short peptides [15]. Traditionally there are two main strategies for the addition of biological cues on surfaces. The first one was based on the use of natural proteins, and the second one was based on the use of simplified artificial versions, that is, short peptides. Both strategies present advantages and disadvantages and there is not a fixed consensus of which is the best solution. For instance, the use of natural proteins is useful because it allows the incorporation of the full potential of the protein. Normally, natural proteins on its native form present synergistic sequences that trigger stronger cell response in a more specific and complex manner. However, natural proteins may present immunogenic problems, purification limitations, batch to batch variations and low functionalization control. Instead, short peptides are non-immunogenic, easier to purify, synthetized in a reproducible manner, and can be grafted in a controlled fashion. Short peptides, however, are normally oversimplified constructs, with lower cell specificity, less effectiveness, weaker stability and cannot encode complex functionalities [16].

As an alternative, a third strategy appeared, which was based on the use of recombinant proteins that were synthetized to gather the advantages of both previous systems, and avoid the major drawbacks [17]. Thus, recombinant proteins, obtained by means of biotechnological tools, started to gain interest. The study of these novel molecules raised high expectations because not only can be design to be non-immunogenic, and include tailored bioactive motifs, but they also can be design to perform complex activities [18, 19]. In such a way, it is possible to obtain smart constructs that show more stability, complexity and simplified purification steps.

The design of these engineered proteins is based on the mimicry of natural proteins. Elastin-like recombinamers (ELRs) are a kind of synthetic biopolymers built from the combination of different modules derived from the natural protein elastin, which confer the desired structural and functional properties to the final construct [20]. ELRs are normally built using the VPGIG amino acidic building block, which mimics the natural non-immunogenic pentapeptide VPGVG from the natural elastin protein [21]. This pentapeptide confers to the synthetic construct the elastic properties of elastin, and can be modified to include other capabilities such as cross-linking sites. Moreover, other modular sequences, such as the integrin-recognized RGD sequence, can be introduced inside the construct to confer to this protein biological functionality [22]. ELRs are also interesting because they can be codified into synthetic genes to be produced by *Escherichia coli* in large amounts, maintaining good control on peptidic sequence and molecular mass, and can be easily purified through their inverse temperature transition properties as well.

In this study, the biofunctionalization of standard PMMA surfaces with ELRs constructs is studied and compared with the use of short peptides and a natural protein (fibronectin). The goal of the study is to investigate the biofunctionalization method of PMMA and analyse and detect the best functionalization strategy in terms of cell–biomaterial interaction, so it can be translated to the final device to improve the interface integration. Surface characterization results and in vitro studies show that the highest cellular attachment efficiency is found on ELR-functionalized PMMA surfaces.

## Materials and Methods

### Materials

PMMA (Acryl) was purchased from GoodFellow (Spain). Short peptides were obtained from Genscript (USA). These peptides were arranged to contain an integrin binding motif (RGDS) and a six-glycine sequence spacer in the amino end. As a negative control, a short peptide with the RGE sequence was used. Active and negative control peptides were named G_6_RGDS and G_6_RGE, respectively. Both peptides were obtained desalted and dehydrated.

ELRs were fabricated using previous protocols [23]. Briefly, ELRs were produced on cultures of *Escherichia coli* strain BLR (DE3) with transferred ELR recombinant gene. ELR production was carried out in a 12-l Applikon fermenter, in Terrific Broth medium with 0.1% of carbenicilin and 0.1% of glucose, at 37°C and pH 7.00. Once the fermentation is finished, cells were collected by centrifugation and lysed by ultrasonication. Cell debris was removed with a centrifugation step. Protein lysate was purified with repeating cycles of cold (4°C) and warm (40°C) centrifugations. During purification steps, 0.5 M sodium chloride solution was used as solvent. Finally, the obtained ELR purified solution was frozen and lyophilized.

The ELR containing the RGD sequence, named HRGD_6_, and the respective ELR control without the RGD sequence, named IK_24_, were encoded with the following amino acidic sequences:

HRGD_6_: MGSSHHHHHSSGLVPRGSHMESLLP[[(VPGIG)_2_(VPGKG)(VPGIG)_2_]_2_AVTGRGDSPASS[(VPGIG)_2_(VPGKG)(VPGIG)_2_]_2_]_6_.

IK_24_: MESLLP (VPGIG VPGIG VPGKG VPGIG VPGIG)_24_.

ELR sequences contained VPGKG modules to dispose lysine residues (K) inside the construct so it can be covalently grafted on the surface.

All other reagents were obtained from Sigma-Aldrich and used without further purification.

### PMMA films preparation

Solvent-casted PMMA thin films were produced by evaporation of PMMA-CHCl_3_ solution (2.5% w/v) into propylene well plates. CHCl_3_ evaporation was carried out in a solvent-saturated atmosphere for 3 days. Obtained solvent-casted films were kept in vacuum under dry conditions until their use.

### Surface functionalization

Surface chemistry was modified through the covalent grafting of different molecules. The functionalization of the different biomolecules was performed following a three-step protocol. First, on the surface preactivation, the PMMA surfaces were reacted with a sodium hydroxide basic solution at 0.5 M for 16 h and gently washed with water. Thus, due to the hydrolysis of the PMMA ester bonds, carboxyl groups were disposed on the surface. Second, PMMA films were reacted with *N*-(3-dimethylaminopropyl)-*N*′-ethylcarbodiimide (EDC) and *N*-hydroxysuccinimide (NHS) at 0.1/0.2 M in PBS (pH 7.4) to obtain active reactive sites. The activation of PMMA surfaces was carried out for 1 h, as it has been observed that this is the average life-time of NHS active sites in aqueous media with a pH 7.4 [24]. Before third step, PMMA films were rinsed with PBS (pH 7.4). Third, activated surfaces were biofunctionalized overnight with selected biomolecule at 100 µg/ml in PBS solution. During this reaction, the amino-groups of grafted biomolecules were used to form a covalent peptidic bond with the activated carboxyls groups from surface. Obtained films were rinsed with water and stored under vacuum-dry conditions until their use.

Additionally, a set of PMMA films were functionalized with same biomolecules by physisorption. In this case, PMMA films were immersed into a PBS solution containing the biomolecule at a concentration of 100 µg/ml and let it to adsorb overnight. Physisorbed surfaces were adopted as controls during the comparative studies for the analysis of the covalent bond effect on molecular stability and cellular response.

### Surface characterization

#### Contact angle

Chemical surface composition affects the physical surface properties. For that reason, the hydrophobicity of PMMA surface was expected to change with each treatment step. Contact angle is a technique sensitive to substrate hydrophobicity that allows a fast qualitative analysis. To observe the changes induced by reactions, PMMA thin films were analysed by the sessile drop method using a goniometer (OCA 15+, Dataphysics, Germany).

#### Time-of-flight secondary ion mass spectroscopy

Time-of-flight secondary ion mass spectroscopy (TOF-SIMS) was used as a complementary technique to analyse the chemical surface changes. TOF-SIMS (TOF-SIMS IV, Ion Tof) is a very sensitive technique used to detect components on the surface. In this study, nitrogenated components coming from the addition of biological molecules were analysed.

#### Surface activation test

Activated carboxyl group availability on surface was studied using an enzyme-linked immunosorbent assay (ELISA) test. Briefly, PMMA films were covalently grafted, as previously described, with solutions containing different proportions of Glycine (Sigma) and biotinylated Amine-PEG (Pierce Protein Biology Products) molecules. Later, treated films were soaked in blocking buffer [PBS + Tween20 0.05% (v/v) + BSA 2% (w/v)] for 1 h at room conditions to clog non-functionalized sites and rinsed three times with rinsing solution [PBS + Tween20 0.05% (v/v)]. Afterward, Streptavidin-HRP (Pierce Protein Biology Products) molecules were conjugated with PEG-biotinylated molecules during 1 h using the blocking buffer as medium and rinsed six times with rinsing solution. At last, ABTS (2,2′-azinobis [3-ethylbenzothiazoline-6-sulfonic acid]-diammonium salt) (Pierce Protein Biology Products) reactive agent was added to carry on the colorimetric assay following the manufacturer protocols.

### In vitro cell studies

#### Cell culture

Mesenchymal stromal cells from rat (rMSC) were collected from bone marrow [25] and used on passages 4–6. Cells were growth in advanced DMEM medium (A-DMEM) containing 15% fetal bovine serum, 1% l-glutamine and 1% penicillin/streptomycin. Cells were expanded until ∼80% surface confluence prior to passage them.

#### Cell attachment

For the cell adhesion study, PMMA films solvent-casted on 24-well plates were functionalized using the previously described protocol. Samples were sterilized using ethanol 70% (v/v) and UV light and left in a sterile hood until they were completely dry. To ensure ethanol removal, samples were gently washed with PBS (pH 7.4). Some samples were blocked using a PBS (pH 7.4) + BSA 6% solution for 1 h and rinsed again with PBS (pH 7.4). Expanded rMSC were collected and seeded at a concentration of ∼5300 cells/cm^2^, that is 10 000 cells per well. Adhesion assay was performed in serum free A-DMEM and cultured for 4 h. Afterwards, samples were rinsed with PBS to remove non-attached cells. Quantification of adhered cells was performed using a Cytotoxicity Detection KitPLUS (LDH) (Roche) on cultured samples previously treated with a lysis buffer (PBS pH 7.4 + Triton X-100 0.01% (v/v)) according to manufacturer protocol. For the analysis of cell morphology, cells adhered to PMMA-functionalized films were fixed with paraformaldehyde 6% during 4 h and their cytoskeleton and nuclei were stained with. rhodamine–phalloidin and DAPI, respectively. Additionally, focal points were marked using the monoclonal anti-vinculin antibody/Alexa Fluor® 488 goat anti-mouse IgG conjugate system. Fluorescence staining was detected with an Eclipse600 upright microscope.

Fluorescence images were also used to calculate the cell aspect ratio (AR) and cell area distribution. AR was described as the relation between the minor and major axes of the smallest ellipse that could fit the cell area. AR and cell area values were obtained through image processing using the ImageJ software [22, 26].

### Statistics

Results were analysed using the *t*-Student test (*P* < 0.05). Experimental values were presented as mean ± standard deviation. Results showing population distributions were presented in box-plot format. In box-plot graphs, central boxes stand for the second and third population quartiles (central 50% of the population distribution) and bars stand for the first and fourth population quartiles (extreme 50% of the population distribution). In box-plot graphs, the plotted population represents the 95% of the measured values. Thus, extremely deviated results were avoided to prevent false maximum and minimum values.

## Results

### Contact angle

PMMA wettability was evaluated at the different procedure steps to evaluate the effect of surface chemical changes (Table 1).

**Table 1.** contact angle of different treated PMMA surfaces

PMMA non-treated	85.8 ± 1.7
PMMA hydrolysed	82.5 ± 1.3
PMMA activated (NHS)	77.4 ± 0.9
PMMA HRGD6 covalent	73.9 ± 1.3
PMMA IK24 covalent	72.9 ± 2.7
PMMA HRGD6 physisorbed	88.3 ± 2.5

Results showed a first wettability increment with the incorporation of NHS molecule, and a second increment with the covalently functionalization of ELRs. Instead, the functionalization of bioactive molecules by physisorption left to same hydrophobic surface than non-treated surfaces.

### Time-of-flight secondary ion mass spectroscopy

**Figure 1.**
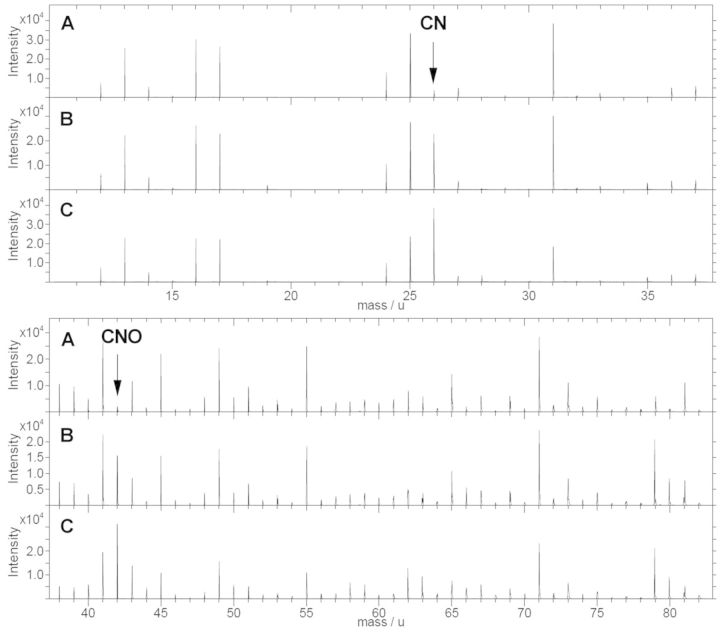
TOF-SIMS negative spectra of (A) non-treated PMMA surface, (B) NHS activated PMMA surface and (C) short peptide-functionalized surface

To validate the covalent functionalization of grafted molecules, the incorporation of nitrogen component into the surfaces was studied by means of TOF-SIMS analysis. Thus, non-treated surfaces, activated surfaces (with grafted NHS molecule) and functionalized surfaces (with short-peptide molecules) were studied (Fig. 1). Only short peptides were used on this study because of their weak physisorbed retention on surface, which ensured complete cleaning of non-cross-linked molecules. Thus, incorporation of nitrogen component could be correlated to covalent bonding with more feasibility.

TOF-SIMS negative spectra showed the apparition of nitrogenized components on activated and functionalized samples but not on non-treated PMMA. Spectra showed new peaks on values associated with CN mass component (26 u) and CNO mass component (42 u).

### Surface activation test

**Figure 2.**
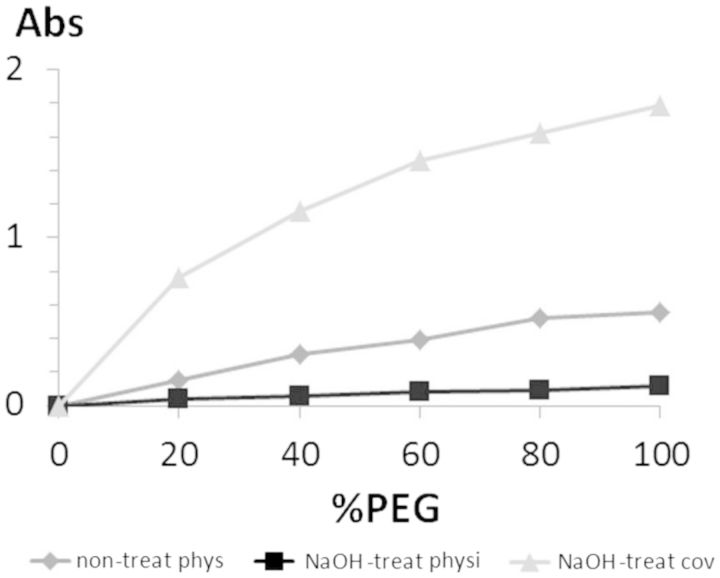
absorbance levels for measured amounts of grafted PEG-b molecule under different conditions: non-treated PMMA surface by physisorption, 24-h NaOH-treated PMMA surface by physisorption and 24-h NaOH-treated PMMA surface by covalent ligation

To evaluate grafting in a semi-quantitative manner, an ELISA was performed. In this case, three different samples were evaluated: a covalently functionalized surface, a physisorbed surface without the NaOH treatment step and a physisorbed surface with the NaOH treatment (Fig. 2). Detection of molecular surface grafting was done for a PEG-biotin molecule, which was used as an analogue of a short peptide. Moreover, all samples were functionalized with different ratios of PEG-biotin/glycine mixtures. The use of different molecular ratios was used to observe different amounts of PEG-b molecule grafted on different treated surfaces and ensure in such a way that the analysis was done inside the technique-sensitive region. Thus, it was possible to ensure that measured values were not given by a saturation signal and they could be compared between them in a semi-quantitatively manner. The physisorbed sample with pre-activation treatment was included to ensure that PEG-b reporter did not adsorb due to ionic forces.

Results showed that covalently functionalized samples presented higher levels of PEG-biotin reporter (3-fold times compared with physisorbed surfaces without pre-treatment step). Moreover, no signal was found on physisorbed samples with NaOH pre-treatment. Some unspecific adsorption of PEG-b reporter was found on physisorbed samples without NaOH pre-treatment, which revealed that weak, but still existing, physisorbed functionalization occurred. Instead, PEG-b levels were almost null for physisorption grafting on pre-treated samples.

### Cell attachment

For the evaluation of cell attachment on PMMA surfaces, different conditions were tested. As negative controls non-treated PMMA (no treat) and BSA-blocked PMMA (BSA) were used, and for the positive control tissue culture polystyrene plate (TCP) and fibronectin-functionalized PMMA (Fn) were used. For the comparative analysis of the role of the RGD sequence and the effect of the biomolecule nature, PMMA surfaces functionalized with ELRs (HRGD_6_) and short peptides (G_6_RGDS), as well as surfaces functionalized with their homologous lacking the RGD sequence (IK_24_ and G_6_RGE), were used. Similarly, surfaces functionalized with same molecules but using physisorption processes were used to study the role of the grafting strategy. Moreover, covalent and physisorbed functionalized surfaces were studied as well under the effect of a BSA-blocking step before cell seeding, to evaluate the non-fouling properties of functionalized surfaces.

**Figure 3.**
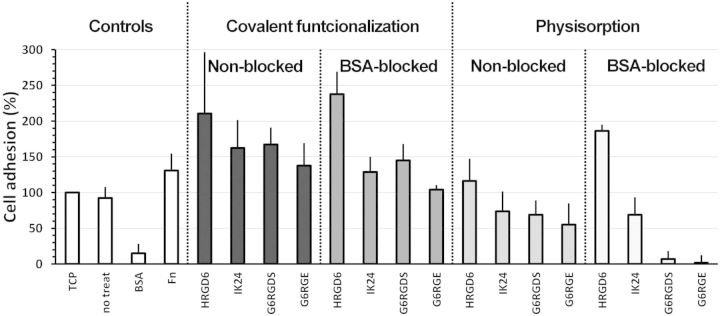
adhesion assay carried out on different functionalization conditions. Cell number was obtained from LHD activity and normalized against TCP positive control. Error bars represented standard deviation

To evaluate short cell response, the number of adhered cells after 4 h of seeding was measured. Comparison of different samples was done using TCP sample as reference, against which all other samples were normalized (Fig. 3).

The adhesion study showed that functionalized PMMA samples enhanced cell adhesion. Indeed, HRGD_6_-functionalized samples presented up to 100–150% more adhered cells than TCP control.

This adhesion study showed the effect of three different factors on cell attachment response. The first factor was the presence or absence of RGD sequence, which positively impacted on the number of adhered cells. Indeed, molecules with encoded RGD sequences (HRGD_6_ and G_6_RGDS) presented more cell adhesion levels than their homologues without the RGD motif (IK_24_ and G_6_RGE). These differences between RGD-containing molecules and RGD-lacking molecules were increased on samples blocked with BSA.

The second factor was the nature of the functionalized molecule. The use of ELRs presented larger adhered cell numbers compared with short peptides. Covalently functionalized HRGD_6_ samples presented between 50% and 80% more adhered cells than covalently functionalized G_6_RGDS samples, whereas physisorbed HRGD_6_ samples presented between 50% and 180% more adhered cells than physisorbed G_6_RGDS samples. Also, results showed that the presence of BSA on the medium affected in a stronger manner the short peptide-functionalized surfaces, whereas had little or no effect on ELR-functionalized surfaces.

The third factor was the effect of the covalent grafting, that is, the grafting stability. In general, when comparing same functionalization conditions, moving from covalently functionalized to physisorption decreases the amount of adhered cells on surface (between a 50% and a 100% difference). Indeed, most covalently functionalized surfaces presented amounts of adhered cells above TCP control, whereas only HRGD_6_ physisorbed surfaces were above the TCP control levels. The other physisorbed surfaces (IK_24_ and short peptides) presented lower amounts of adhered cells than TCP. This effect was found to be stronger on peptide-functionalized surfaces.

The effect of covalent bond was also noticed when BSA protein was introduced on the media, which competed with already adhered molecules for the unspecific adsorption on surface. In this case, the presence of covalent bond enhanced the amount of adhered cells between a 50% and a 100% more, depending on the functionalization conditions. Also it was observed that the presence of covalent bonding was critical for the short peptide engraftment, as almost no cellular adhesion was found on physisorbed short peptides with blocking-BSA.

Cell adhesion strength was also evaluated by means of cell morphology. Thus, the cell shape was observed by fluorescence microscopy and classified using quantitative shape descriptors. During cell morphology studies, surfaces with physisorbed short peptides were not considered, since they presented not enough adhered cells for the statistic study. Then, AR was calculated from processed fluorescence images and displayed on Table 2. Median values were represented instead of average values because population was distributed on a non-Gaussian shape.

**Table 2.** AR of cells adhered on different functionalized surfaces

	Controls			Physisorbed		Covalent			
	No treat	Fn	TCP	HRGD6	IK24	HRGD6	IK24	G6RGDS	G6RGE
BSA-blocked PMMA	1.27	1.61	1.47	1.53	1.29	1.54	1.54	1.49	1.47
Non-blocked PMMA	1.35	1.49	1.47	1.69	1.41	1.56	1.51	1.45	1.36

Using results as a reference, values contained between 1.27 and 1.35 (negative controls) were used to describe round and small cell shape (weak cell adhesion). Instead, values contained between 1.47 and 1.61 (positive controls) were used to describe elongated cell shape (advanced cell adhesion state). Values placed in between these two regions represented intermediate adhesion states. This permitted to observe that, in general, covalent functionalization presented the most advanced cell adhesion states after 4 h of seeding, together with physisorbed HRGD_6_ samples. Instead, surfaces with physisorbed IK_24_ and short peptides showed, in general, an intermediated cell adhesion state, or a poor adhesion state (star-like or round cell shapes, respectively).

**Figure 4.**
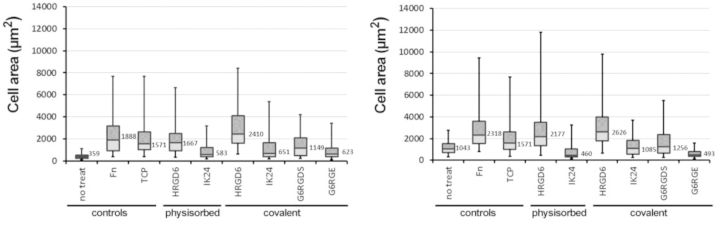
representation of the cell area distribution of different functionalization conditions on a box-plot graph. The square boxes represented the second and third quartiles of the distribution (central 50% of the population) and the bars represented the first and fourth quartiles of the distribution (lateral 50% of the population). The 0.25% of the population found on both extremes of the distribution was excluded from the graph to avoid false maximum and minimum values. TCP positive control (without blocking with BSA) is represented on both graphs as a reference. Numerical values represented the median values of the distribution

To complement cell morphology study, spread cell area was analysed from different sets of fluorescence micrographies. Results were represented in a box-plot graph to observe the cell population distribution (Fig. 4).

Cell spread area results showed that larger areas were found on RGD-containing ELRs (HRGD_6_)-functionalized surfaces, especially on covalently functionalized surfaces, which presented larger cell areas than positive controls. Instead, short peptides and IK_24_-functionalized surfaces presented smaller cell areas, which represented a weaker cell attachment. Interestingly, it was possible to observe that molecules presenting RGD sequence had larger cell areas on BSA-blocked surfaces than non-blocked surfaces, whereas molecules without the RGD sequence, in general, presented an opposite behaviour (smaller cell areas with the presence of BSA protein). When crossing the cell area information with the adhered cell number results, it was interesting to observe that non-treated samples, despite adhering around ∼80% of cells, they presented a completely round cell shape, which was a sign of a poor adhesion state. Similar behaviour was found on surfaces functionalized with IK_24_ and G_6_RGE molecules.

**Figure 5.**
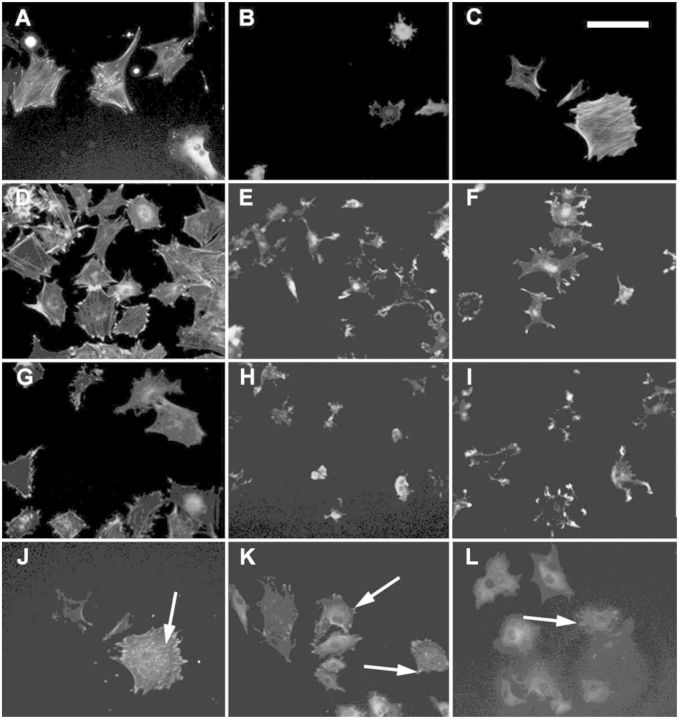
immunofluoresence pictures of cells cultured on (A) TCP, (B) non-treated PMMA, (C) PMMA-Fn, (D) PMMA-HRGD_6_ covalently functionalized, (E) PMMA-IK_24_ covalently functionalized, (F) PMMA-G_6_RGD covalently functionalized, (G) PMMA-HRGD_6_ physisorbed, (H) PMMA-IK_24_ physisorbed, (I) PMMA-G_6_RGE covalently functionalized, (J) PMMA-HRGD_6_ covalently functionalized, (K) PMMA-HRGD_6_ physisorbed and (L) PMMA-G_6_RGD covalently functionalized. Images (A)–(I) represent cells with stained actin cytoskeleton. Images (J)–(L) represent cells with stained vinculin. White arrows on images (J)–(L) show the presence of focal points. Scale bar = 100 µm

The direct observation of cell morphology by fluorescence was in concordance with the numerical values calculated over cell populations (Fig. 5). Thus, it was possible to observe round and small cell shapes on non-treated samples and IK_24_-physisorbed samples, whereas short peptides and IK_24_ covalent samples presented a star-like cell shape. Instead, positive controls and HGRD_6_-functionalized samples (both covalent and physisorbed) were the only conditions presenting large cell areas with a clear cytoskeleton organization with well-formed actin stress fibres. Interestingly, when observing the formation of focal points, it was possible to observe that they were only present on samples functionalized with molecules containing RGD sequences. Moreover, it was possible to see that, generally, positive controls and covalently functionalized HRGD_6_ surfaces presented cells with focal points along the entire cell body base, whereas physisorbed HRGD_6_- and G_6_RGDS-functionalized surfaces only presented focal points on the periphery of the cell body, that is the *lamellipodia*.

## Discussion

A key aspect on tissue engineering is the integration of implants on hosting tissue, which is tightly correlated with a proper cell–material interaction on the interface. In this study, the interaction between biofunctionalized PMMA surfaces and cells is investigated. A comparative analysis has been carried out involving commonly used strategies, such as short RGD peptides and fibronectin, faced to a novel strategy based on the use of genetically modified protein, that is ELRs.

To validate the grafting of functionalized molecules, three different techniques were used. The first one, contact angle, was used to detect the chemical changes on surface occurred due to treatment steps. Hydrolysis, activation and functionalization changed the composition of the functional groups shown on surface, and consequently the surface wettability. Surface wettability is correlated with the hydrophobicity of surface, which is an important parameter due to its role on protein non-specific adhesion and cellular attachment. Normally, hydrophobic surface adsorbs unfolded proteins in an unspecific manner, and presents low levels of cellular attachment [14, 27]. Indeed, adsorption of proteins on hydrophobic surfaces tends to denature their native conformation, and, because it is unspecific, tends to accumulate high levels of adsorbed proteins from serum like BSA protein, which prevents a correct cell–material interaction and induces the formation of a cystic layer [28]. Contact angle results showed that PMMA non-treated surfaces were hydrophobic (85.8°), whereas successive treatments lowered it until 73.9°. The decrease of the contact angle revealed in an easy manner the chemical changes occurring at the interface, and also warned about the interactions of proteins and cells against the surface. For instance, the high hydrophobicity observed on all treated and non-treated surfaces defined these surfaces as highly attractive for BSA unspecific adsorption, whereas lower contact angles found on functionalized surfaces defined them as more cell appealing compared with non-treated surfaces.

The second technique was used to clearly corroborate the activation of PMMA surfaces and the molecular engraftment by means of covalent ligation. TOF-SIMS was used to analyse non-treated surfaces, activated surfaces and functionalized surfaces with short peptides. Only short peptides were used because it was easier to remove non-covalently grafted molecules and correlate the apparition of nitrogenized components with cross-linked engraftment. Thus, it was possible to observe the apparition of new peaks on the negative spectra on activated and functionalized surfaces, which were correlated with the CN and CNO mass component. The apparition of nitrogenized components was caused by the incorporation of NHS molecules and short peptides, which proved their grafting onto surfaces. Moreover, it was possible to observe that peak intensities were higher on surfaces functionalized with short peptides than on activated surfaces, which agrees with the fact that a single peptide molecule contains more nitrogen atoms than an NHS molecule.

The third technique was based on an ELISA test that used a PEG-biotin probe grafted under different conditions. The PEG-b probe was used as a reporter detected by means of biotin–streptavidin affinity through the ELISA test. PEG-b probe is a small molecule used as an analogue of a short peptide that can be easily removed by washing steps, so only strong grafted molecules were measured. PEG-b was grafted by physisorption on a non-treated surface and a 24-h NaOH-treated surface, and through covalent bonding on a 24-h NaOH-treated surface. Samples with physisorbed molecules on 24-h NaOH-treated surfaces were used as a negative control to ensure that no molecules were adsorbed due to unspecific electrostatic interactions. In the assay, different ratios of PEG-b/glycine mixtures were functionalized. These mixtures were used as a control, so that detection of different quantities of grafted PEG-b molecules was a guarantee of assay sensibility. Results showed that covalently functionalized sample signal was 3-fold times more than physisorbed ones, which were found in much less quantities. Thus, it was indirectly proved the occurrence of the covalent functionalization. Negative control ensured that the probe did not adsorb on treated surfaces, so the entire measured signal from covalent sample belonged to cross-linked molecules. It was important to notice that a certain extent of unspecific adsorption occurred on non-treated samples but not on NaOH-treated surfaces. This is important since it showed the low efficiency of functionalization processes by means of adsorption of small molecules.

Finally, to see the effect of the different functionalization strategies on cell response, cell adhesion was evaluated. Thus, the number of adhered cells, cell morphology (elongation and cell area), cytoskeleton organization and focal point formation were assessed. Moreover, the BSA protein was used as competing agent to see the stability of grafted molecules and analyse the unspecific adhesion of albumin. Normally, on a system with more than one protein, the different molecules compete to adsorb on surface. In this way, it was possible to analyse if albumin, a protein found in large quantities on serum, could displace grafted proteins, or adsorb over them, blocking their biological capacities.

Cellular results concluded that the studied molecules were affected by BSA adsorption when they were grafted by physisorption. Indeed, short peptides and IK_24_-functionalized samples had reduced amounts of adhered cells when BSA was added on the media, especially under physisorption conditions. This was explained because these molecules were partially removed or blocked by the unspecific adsorption of BSA protein. However, HRGD_6_-functionalized samples were not affected by this interference and they maintained high amounts of adhered cells. This stability of HRGD_6_-functionalized molecule was correlated to the non-fouling capability of ELRs [25], which prevented the BSA unspecific adsorption, and therefore ELR displacement or masking. BSA adsorption prevention is known to be important to have good tissue–implant integration, as BSA adsorption tends to trigger cystic encapsulation.

ELR covalent ligation not only showed higher amounts of adhered cells but also showed a more developed adhesion state. This was observed on cell morphology analysis, where HRGD_6_ samples showed higher AR (more elongated cells). Moreover, highest cell areas were observed on HRGD_6_ covalently functionalized, which means that cells could spread more when they had adhesive sequences strongly anchored on surface. Instead, physisorbed HRGD_6_ showed smaller cell areas than their covalent homologous, which could be caused by the absence of fixed anchorage points that difficult the self-pulling and stretching of adhered cells. On the other hand, short peptides showed much less spread cell areas. This could be explained because ELRs molecules have certain degree of malleability because of their larger size and cells can stretch them to create focal points, whereas short peptides, because of their shorter nature, present less degree of malleability and focal point formation is less flexible [22]. Interestingly, results also showed that, for those samples functionalized with RGD-containing molecules, cell areas found on PMMA-blocked surfaces were higher than cell areas found on PMMA-non-blocked surfaces. Instead, samples functionalized with RGD-lacking molecules did the contrary. This effect could be explained because BSA protein blocked unspecific cell adhesion regions, which promoted RGD–integrin interaction and, therefore, formation of stronger anchorage and cell spreading on surfaces functionalized with RGD-containing sequences. Equally important was the direct observation of cell morphology and cytoskeleton organization by fluorescence imaging. There, it was possible to observe that, except HRDG_6_-functionalized surfaces, all other samples presented low cell adherence (round shape), or intermediate cell adherence (star shape), and only functionalized surfaces presented focal points on all cell base, which resulted from a good cell anchorage. Focal points found on the vertex of *lamellipodia* of star-like cell shapes were a sign of the stretching of the on-going spreading of cells. Thus, G_6_RGDS-functionalized samples and HRGD_6_-physisorbed samples were defined as intermediate adhesion state, whereas covalent HRGD_6_ was defined as advanced cell adhesion state.

## Conclusions

The biofunctionalization of PMMA surfaces using ELRs has been analysed in this article. ELR-engineered proteins have shown to be a good alternative to short peptides and natural proteins, which can enhance surface properties while avoiding problems such as immunogenicity, purification, over-simplification, and so on. Moreover, ELRs have shown better performance compared with both other strategies. ELRs not only have shown more cell adhesion capacity, but, because of their non-fouling properties, their biological activity is not diminished when BSA is present on the media. This property permits to ELRs to improve tissue–biomaterial interface and promote a better implant integration. The study also showed that better results are found when molecules are covalently grafted to surface, as adhered cells can spread better and have stronger adhesion points.

Because of the successful performance of ELRs, these molecules can be used for the functionalization of PMMA surfaces to obtain stronger and faster cell responses on tissue–biomaterial interface, thus, promoting better implant integration than short peptides functionalization.
